# Predictive Factors Associated With Survival in Periampullary Cancers Following Pancreaticoduodenectomy: A Retrospective Analysis

**DOI:** 10.7759/cureus.50607

**Published:** 2023-12-15

**Authors:** Narendra Maharjan, Ramesh Singh Bhandari, Paleswan Joshi Lakhey

**Affiliations:** 1 Department of Surgical Gastroenterology, Tribhuvan University Teaching Hospital, Maharajgunj Medical Campus, Institute of Medicine, Kathmandu, NPL

**Keywords:** pancreatic carcinoma, ampullary carcinoma, predictive factor, pancreaticoduodenectomy, periampullary carcinoma

## Abstract

Background

Periampullary cancers arise from four different anatomical sites and are in close proximity. But they have different survival outcomes. There are various clinicopathological factors associated with survival after pancreaticoduodenectomy done for periampullary cancers. So, we aimed to identify the predictive factors associated with poor survival in periampullary cancers at Tribhuvan University Teaching Hospital, Kathmandu, Nepal.

Methods

We analyzed the medical records of patients who underwent pancreaticoduodenectomy (PD) at Tribhuvan University Teaching Hospital, Kathmandu, from April 2004 to May 2014. Demography, clinicopathological features, and survival outcomes were analyzed retrospectively.

Results

This study included 61 patients. The mean age of patients was 56.2 ± 14.2 years, and there was a male preponderance (M:F = 1.4). The median survival of all patients was 24 months. Non-pancreatic periampullary cancer patients had better median survival as compared to pancreatic cancer patients (24 vs. 8 months, p = 0.03). The presence of lymphovascular invasion (LVI), peripheral invasion (PNI), nodal involvement, and a higher lymph node ratio (LNR) were associated with poor median survival. However, perineural invasion was the only factor associated with poor survival in multivariate analysis.

Conclusion

The presence of perineural invasion is associated with poor survival outcomes in patients with periampullary cancer following pancreaticoduodenectomy. Also, carcinoma of the head of the pancreas has poor survival as compared to other periampullary cancers.

## Introduction

This article was previously presented as an e-poster at the 15th IHPBA World Congress in New York City in March 2022 and was published online as an abstract in October 2022 in the HPB journal.

Periampullary cancer includes neoplasms arising from four different anatomical sites with proximity to the major duodenal papilla: head of pancreas, ampulla of Vater, distal common bile duct (CBD), and periampullary duodenum [1]. Surgical resection (pancreaticoduodenectomy) is the standard treatment modality for resectable periampullary cancer that offers a chance for a cure. This surgical procedure has evolved over a period of time since it was first described by Codvilla in 1898, but it is still associated with high morbidity (40%), though mortality has decreased to less than 5% [2]. Although these tumors are in close proximity anatomically, they have different survival outcomes. Non-pancreatic periampullary cancers have a more favorable five-year overall survival (25% to 75%) as compared to pancreatic head cancers (0% to 20%) [1].

Various clinicopathological factors, like lymphovascular invasion, perineural invasion, resection margin, and lymph node involvement, have been studied to determine the survival outcome after pancreaticoduodenectomy for periampullary cancers [3,4]. These factors can help in determining the prognosis of a patient as well as in treatment planning. So, this study aimed to identify the predictive factors associated with poor survival in periampullary cancers following pancreaticoduodenectomy at Tribhuvan University Teaching Hospital, Kathmandu, Nepal.

## Materials and methods

We retrospectively analyzed records of patients who underwent pancreaticoduodenectomy (PD) at Tribhuvan University Teaching Hospital, Kathmandu, Nepal, from April 2004 to May 2014. The data was updated until April 2021. The staging of the disease was done according to the 8th edition of the American Joint Committee on Cancer (AJCC). Patients who had periampullary carcinomas (ampullary carcinoma, distal cholangiocarcinoma, pancreatic head carcinoma, and duodenal adenocarcinoma) in histopathological examination were included.

Diagnosis and clinical staging of the disease were done by cross-sectional imaging like contrast-enhanced computed tomography (CECT) of the abdomen and pelvis, chest X-rays, and duodenoscopy. Then the patients with resectable disease were subjected to open PD. Data on demographics, primary diagnosis, histopathological diagnosis, pathological staging, and long-term outcome in terms of survival were retrieved from medical records and analyzed.

The primary endpoint of the study was overall survival. The overall survival was calculated as the duration from the date of diagnosis until the last follow-up or death. The lymph node ratio (LNR) was defined as the number of lymph nodes with metastases divided by the total number of excised lymph nodes. The microscopic resection margin was considered positive when the tumor involved less than 2mm from the margin.

The study protocol was reviewed and approved by the Institutional Review Board at Tribhuvan University Teaching Hospital. Descriptive statistics like median, frequency, and percentage were used for categorical variables. The survival outcome was analyzed by the Kaplan-Meier method.

The prognostic variables of overall survival selected were lymphovascular invasion (LVI), perineural invasion (PNI), resection margin, lymph node positivity, and lymph node ratio. Univariate and multivariate analyses of prognostic variables for overall survival were done using Cox regression analysis. Factors that were found to be significant in univariate analysis were included in multivariate analysis. Statistical analysis was performed using IBM Corp. Released 2019. IBM SPSS Statistics for Windows, Version 26.0. Armonk, NY: IBM Corp.

## Results

**Figure 1:**
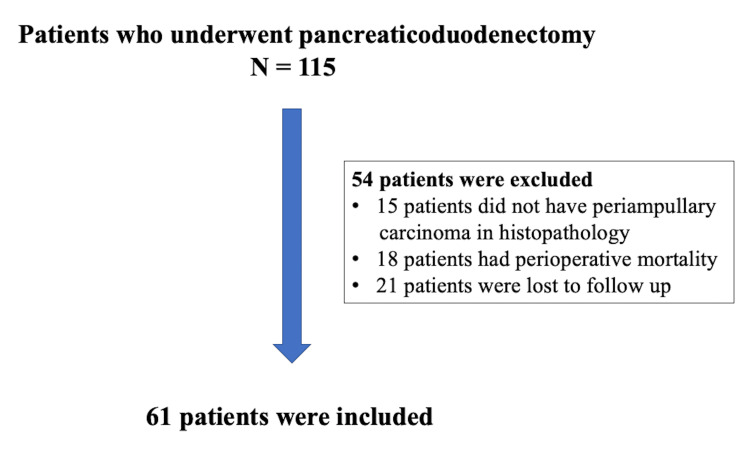
Patients included in the study n: number

There were 115 patients who underwent PD from April 2004 to May 2014. Among them, 54 patients were excluded from the study for various reasons, as shown in Figure 1. Thus, the study included 61 patients. 

The median duration of follow-up was 12 months (3 to 180 months). Demography and clinic-pathological features are shown in Table 1.

**Table 1:** Demography and clinicopathological features. SD: Standard deviation, LVI: Lymphovascular invasion, PNI: Perineural invasion.

Variable		Value
Age, in years, Mean ± SD		56.2 ± 14.2
Sex (male), n (%)		36 (59)
Jaundice at presentation, n (%)		54 (88.5)
Length of hospital stay, mean		16.8±10.6 days
Pathological subtypes	Ampullary carcinoma	42 (68.5%)
	Distal cholangiocarcinoma	7 (11.5%)
	Pancreatic adenocarcinoma	6 (10%)
	Duodenal adenocarcinoma	6 (10%)
Pathological staging	0	1 (1.6%)
	IA	7(11.5%)
	IB	18 (29.5%)
	IIA	9 (14.8%)
	IIB	22 (36.1%)
	III	4 (6.6%)
Patients with lymph node positive (N1)		21 (34.4%)
Median number of lymph node yield		6 (0-35)
LVI present		17 (28%)
PNI present		16 (25%)
Resection margin positive		6 (10%)

**Figure 2:**
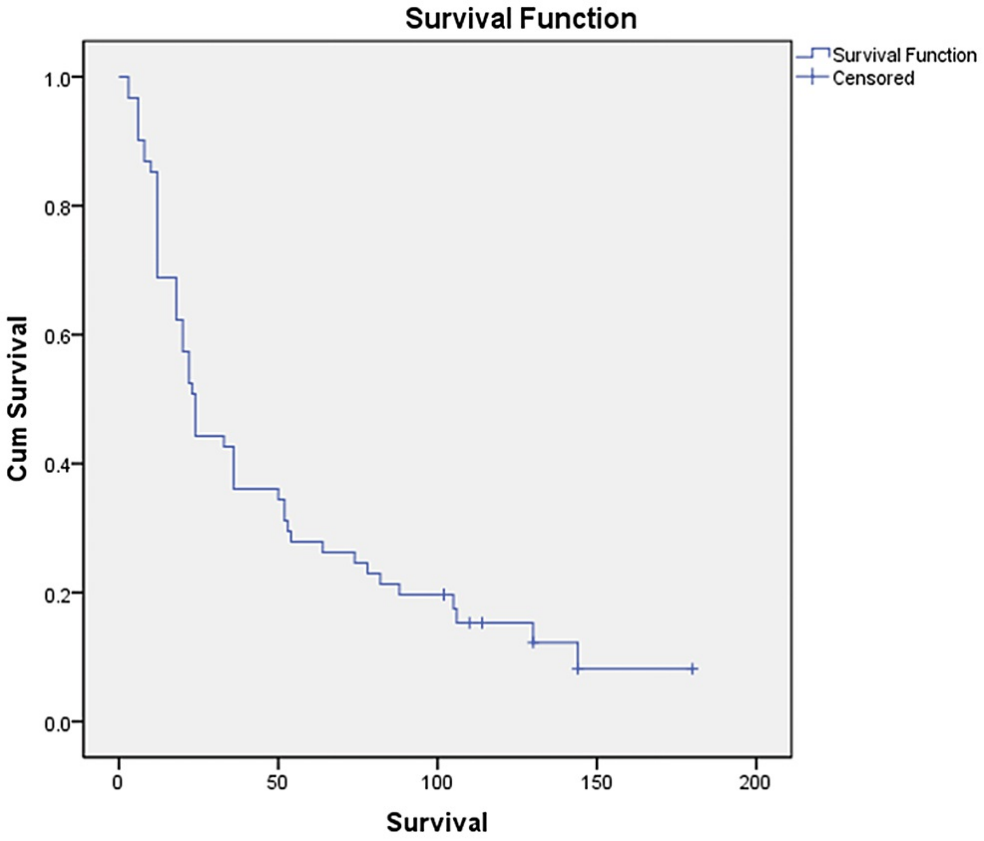
Kaplan-Meier plot of the survival of patients with periampullary cancer.

The mean age of patients was 56.2 ± 14.2 years, and there was a male preponderance (M:F = 1.4). Most of the patients (88.5%) had jaundice at the time of presentation, and ampullary carcinoma (68.5%) was the most common pathology among the periampullary cancers. Ten percent of the patients had a microscopic resection margin positive (R1). The median survival of all patients was 24 ± 44.3 months (Figure 2).

**Figure 3:**
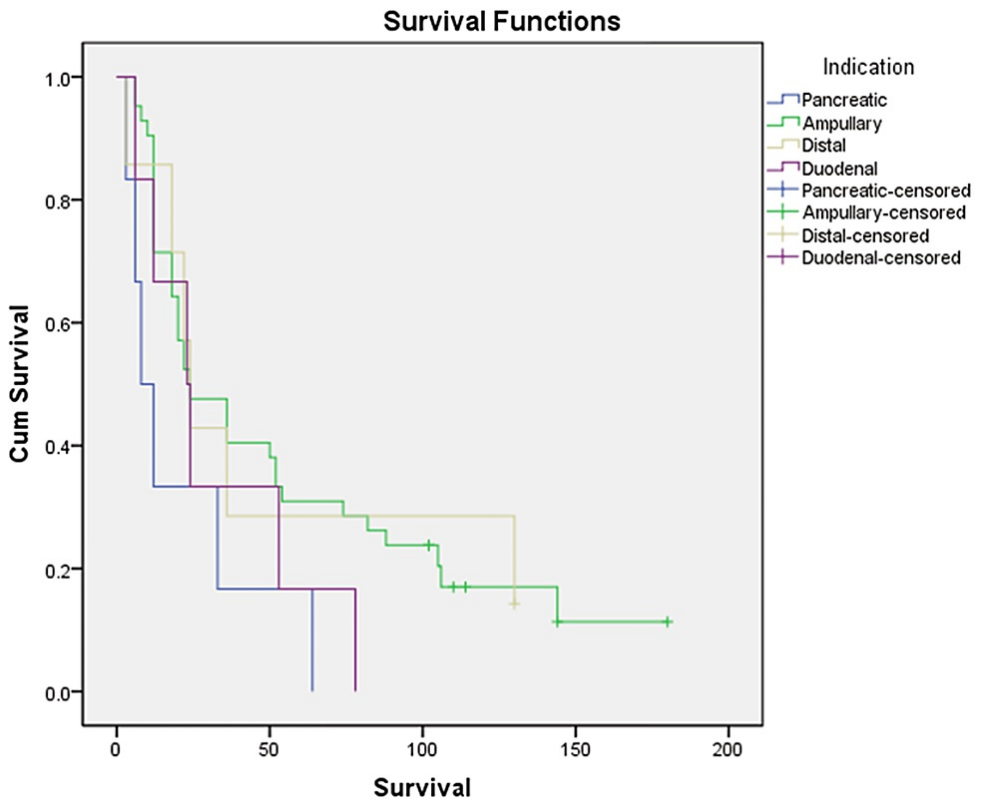
Comparison of median survival among various periampullary carcinomas.

The median survival of patients with pancreatic carcinoma, ampullary carcinoma, distal cholangiocarcinoma, and duodenal adenocarcinoma was 8, 24, 24, and 23 months, respectively (Figure 3).

**Figure 4:**
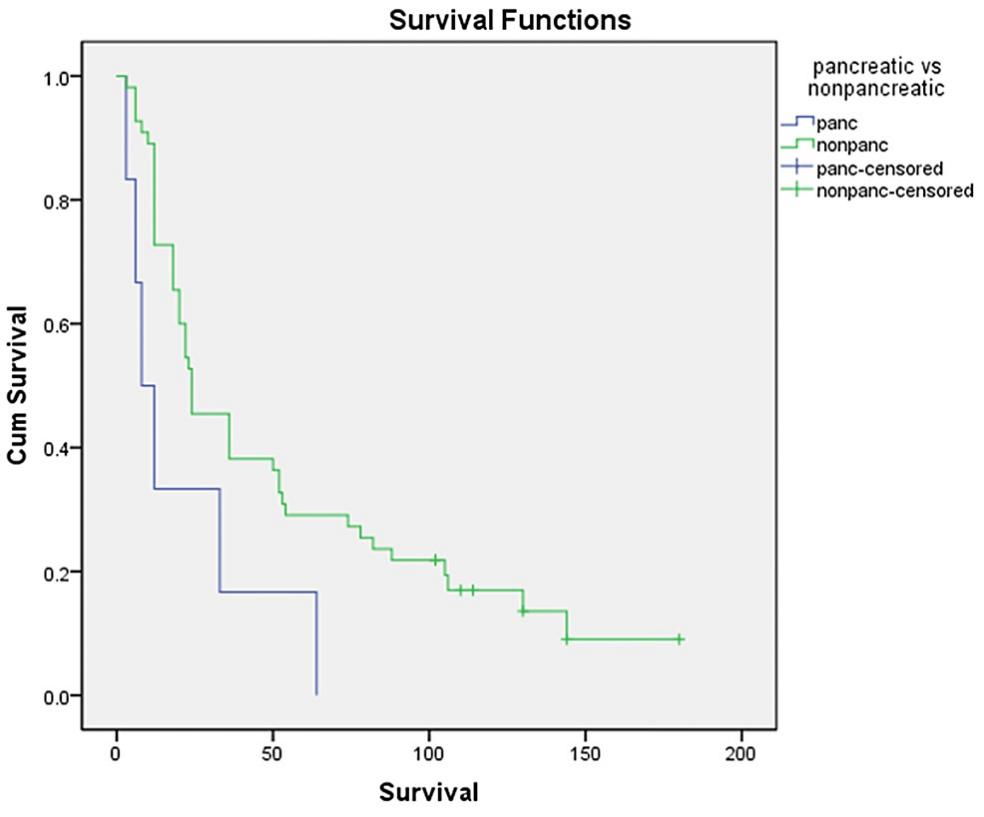
Comparison of median survival between pancreatic and non-pancreatic carcinoma.

Non-pancreatic periampullary cancer patients had better median survival as compared to pancreatic cancer patients (24 vs. 8 months, p = 0.03), as shown in Figure 4.

The presence of lymphovascular invasion (LVI), perineural invasion (PNI), nodal involvement, and a higher lymph node ratio (LNR) was associated with poor median survival (Table 2).

**Table 2:** Prognostic factors and median survival (Kaplan-Meier test).

Prognostic factors		Median survival (months)	p value
Age	<55	22	0.566
	≥55	24	
Gender	Male	22	0.329
	Female	36	
Pathological stage	0	64	0.010
	IA	74	
	IB	36	
	IIA	33	
	IIB	12	
	III	6	
Pathological subtype	Pancreatic	8	0.03
	Non-pancreatic	24	
Lymphovascular invasion	+ve	12	0.040
	-ve	33	
Perineural involvement	+ve	12	0.001
	-ve	33	
Resection margin	R0	24	0.056
	R1	8	
Nodal involvement	N0	36	0.009
	N1	12	
Lymph node ratio	<0.2	36	0.001
	>0.2	12	

However, perineural invasion was the only factor associated with poor survival in multivariate analysis (Table 3).

**Table 3:** Prognostic factors and median survival (multivariate Cox regression analysis).

Prognostic Factors	p-value	Hazard ratio	95% Confidence interval	
			Lower	Upper
Pathological subtype	0.35	0.48	0.10	2.26
Lymphovascular invasion	0.63	1.29	0.44	3.77
Perineural involvement	0.01	3.41	1.26	9.26
Nodal Involvement	0.27	2.58	0.48	13.85
Lymph node ratio	0.30	0.52	0.15	1.82
Pathological stage	0.73	1.60	0.10	24.66

## Discussion

Pancreaticoduodenectomy is the standard treatment for periampullary cancers. Postoperative complications and mortality have decreased with the advent of better surgical techniques and perioperative care. However, the long-term survival outcome is still low, and the five-year survival rate reported varied widely [4]. It ranged from 34.9% to 54% five-year survival [5,6].

El Nakeeb et al. showed that five-year survival was 20.6%, and median survival was 34 months [7]. They reported the worst prognosis in patients with pancreatic head adenocarcinoma and a better prognosis in patients with ampullary and duodenal adenocarcinoma. In our cohort, the median survival of the patients was 24 months, and pancreatic head cancer had a poor survival outcome as compared to other periampullary cancers. This may be because pancreatic cancer is known to be a biologically more aggressive tumor and has a higher incidence of nodal spread, perineural invasion, and lymphovascular invasion [8].

Histopathological characters like lymphovascular invasion, lymph node involvement, and perineural invasion have been shown to be predictors of survival. It is postulated that perineural invasion may be responsible for local treatment failure because tumors can grow along the nerve supplying the pancreas and then to the periarterial neural plexus. Similarly, lymphovascular invasion is considered responsible for regional or distant lymph node metastasis as well as solid organ metastasis like liver and lungs [9]. Chen JW et al. demonstrated that five-year survival was 77% in patients negative for lymphovascular invasion and perineural invasion, while 15% in patients positive for both factors [9]. In our study, both of these factors were significant predictors of survival in univariate analysis, but multivariate analysis showed perineural invasion as the only significant factor.

The LNR was first reported to be related to the prognosis of gastric carcinoma by the Japanese. Then, it was implemented in other gastrointestinal cancers. LNR has been suggested as a predictor of survival in patients with periampullary carcinoma [10,11]. However, LNR was not a significant predictor of survival in our study.

The resection margin after pancreaticoduodenectomy included all margins (i.e., anterior, posterior, pancreatic neck, and portal vein margins). There are mixed results regarding the effect of a positive resection margin (R1) on survival outcomes. Some studies showed that positive resection margin (R1) was a significant predictor of poor survival outcome [9,12], while other studies did not [13,14]. Our study is consistent with the later. This may be due to a lack of standard pathological examination and controversy regarding the definition of microscopic margin involvement used by the various studies.

The AJCC classification has been shown to have prognostic value in most of the malignancies, including those of pancreatic carcinomas [15,16]. But, in this study, AJCC pathological stage was not a predictor of survival in multivariate analysis. Perineural invasion was shown to be an independently significant prognostic factor for survival in this study as well as in other studies [9,17]. Despite this fact, PNI has not been incorporated into the AJCC staging system. These studies strongly claim the inclusion of this parameter in any postoperative staging system. This study has some limitations. It is a retrospective study conducted in a single center with a small sample size.

## Conclusions

The presence of perineural invasion is associated with poor survival outcomes in periampullary cancer patients following pancreaticoduodenectomy. Also, pancreatic cancer has poor survival as compared to other periampullary cancers.
